# Basophil Activation Test Provides Additional Diagnostic Value Beyond Ara h 2–Specific IgE Testing in Peanut Allergic Adults

**DOI:** 10.1111/cea.70386

**Published:** 2026-07-02

**Authors:** J. S. H. Schaapherder, K. C. M. Verhoeckx, E. F. Knol, D. Hamann, B. A. Wevers, A. C. Knulst, J. J. W. Kuiper, T. T. M. Le

**Affiliations:** ^1^ Department of Dermatology/Allergology University Medical Center Utrecht, University Utrecht Utrecht the Netherlands; ^2^ Center for Translational Immunology University Medical Center Utrecht, University Utrecht Utrecht the Netherlands; ^3^ Central Diagnostic Laboratory University Medical Center Utrecht Utrecht the Netherlands; ^4^ Department of Ophthalmology University Medical Center Utrecht Utrecht the Netherlands

## Abstract

- A positive basophil activation test (BAT) confirms peanut allergy in adults with 100% PPV.
- Combining Ara h 2‐sIgE and BAT improves diagnostic accuracy, reducing the need for food challenges.

To the Editor,

For the diagnosis of peanut allergy, the basophil activation test (BAT) has a high positive predictive value (PPV) and negative predictive value (NPV) in children, and is suggested to be used in combination with specific‐IgE (sIgE) against Ara h 2 [1, 2]. Studies investigating the diagnostic value of the BAT in adults are scarce. In adults, two previous studies from our centre showed that peanut allergy could be predicted correctly in around one‐third of patients using a 100% PPV cut‐off value of ≥ 1.75 kU/L for Ara h 2 [3, 4]. In this diagnostic accuracy study, the primary aim was to prospectively investigate the diagnostic value of the BAT in peanut allergic adults. The secondary aim was to evaluate whether sequential testing using Ara h 2‐specific IgE at a cut‐off of ≥ 1.75 kU/L followed by BAT improves the sensitivity for detecting peanut allergy.

Forty‐four adults (mean age 30 years, range 18–67 years) were prospectively and consecutively enrolled, meaning every eligible patient scheduled for an oral food challenge (OFC) at the University Medical Center Utrecht in the Netherlands for a suspected peanut allergy, was included without selection bias. All patients provided written informed consent and the study was approved by the local ethics committee (protocol number: B23‐233). Serum sIgE‐Ara h 2 was measured using ImmunoCAP (Thermo Fisher Scientific, Uppsala, Sweden). BAT was performed against peanut extract, recombinant Ara h 2, and Ara h 6 using a commercially available peanut BAT (Buhlmann, Schönenbuch, Switzerland) within 24 h after blood withdrawal. The BAT was considered not interpretable if < 200 basophils were measured, the patient was a non‐releaser (< 15% CD63+ basophils upon treatment with anti‐FcεRI) or a spontaneous releaser (> 15% CD63+ basophils upon treatment with buffer). The BAT was considered positive if one of the tested conditions (4/20/100 ng/mL peanut extract, 0.8/4/20 ng/mL Ara h 2, or 0.8/4/20 ng/mL Ara h 6) had > 15% CD63^+^ basophils. The guidelines recommend a cut‐off threshold of 15% for extracts and 10% for individual components; however, a uniform threshold of 15% was applied in this study to maximise specificity. A peanut OFC served as the reference standard for all analyses, and was performed according to the PRACTALL guideline [5]. Both laboratory personnel analysing the BAT and clinical personnel interpreting OFC were blinded. The dataset is available in DataverseNL [6].

The BAT was evaluated for all 44 patients, except for 7 patients who were BAT non‐responders. Of these, 37 patients, 23 adults had OFC‐confirmed peanut allergy. The BAT was positive in 12 out of the 23 peanut allergic patients, and none of the cases with a negative OFC had a positive BAT. This resulted in a 52% sensitivity, 100% specificity, 56% NPV, and 100% PPV of the BAT. All patients with a positive BAT reacted to peanut extract. Four out of 12 patients showed no reactivity to Ara h 2 and/or Ara h 6.

We further evaluated whether the BAT has an additional diagnostic value when tested after sIgE against Ara h 2. A sequential diagnostic approach using Ara h 2‐sIgE combined with BAT is shown in Figure 1. Out of the 44 patients with suspected peanut allergy, 11 patients (25%) were correctly diagnosed as OFC‐positive by using the previously found Ara h 2 cutoff level of ≥ 1.75 kU/L [3]. Of the remaining 33 suspected peanut allergic patients with sIgE against Ara h 2 < 1.75 kU/L, 29 patients were IgE‐mediated releasers and had > 200 basophils and thus were included in the BAT analysis. Six out of those 29 cases were BAT‐positive and all 6 were OFC‐positive, resulting in a 100% PPV for the BAT. Among the 23 cases with a negative BAT, 14 cases were correctly OFC‐negative, resulting in a NPV of 61% for the BAT. Overall, of the 44 patients, the combination of Ara h 2‐sIgE testing and the BAT led to a 61% sensitivity, 100% specificity, 59% NPV and 100% PPV.

**FIGURE 1:**
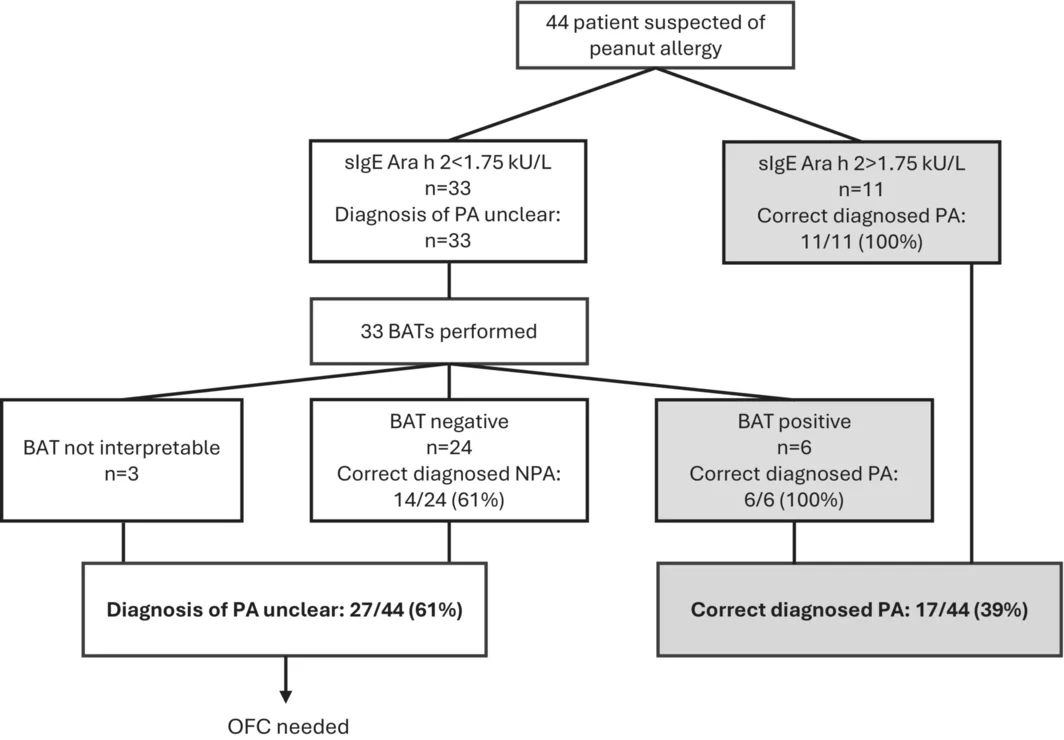
Diagnostic flow chart of the combination of component‐resolved diagnostics for Ara h 2 and the BAT. NPA, not peanut allergic; OFC, oral food challenge; PA, peanut allergy.

Studies evaluating the performance of the BAT in adults are scarce. In this study, we showed that a positive peanut BAT result reliably confirmed peanut allergy in adults with a PPV of 100%. However, a negative BAT cannot exclude peanut allergy. Rentzos and coworkers found a higher sensitivity but lower specificity of the peanut BAT in adults compared to our study (sensitivity: 79% vs. 52% and specificity: 86% vs. 100%) [7]. A possible explanation for this difference is that they determined an optimal cutoff level of the BAT, while we considered the BAT as a dichotomous outcome, as recommended by the manufacturer. In addition, they selectively included anaphylactic peanut allergic adults who did not undergo a food challenge because of the severity of the reaction, while we prospectively and unbiased included all consecutive patients who underwent a peanut food challenge in routine care because of a suspected peanut allergy.

To our knowledge, this is the first study showing that sequential testing of Ara h 2 and BAT improves the diagnostic value in adults. Using Ara h 2‐sIgE alone, 25% of patients could be correctly diagnosed as peanut allergic with a 100% PPV. By subsequently applying the peanut BAT in patients with Ara h 2 levels < 1.75 kU/L, the proportion of correctly diagnosed peanut allergic patients increased to 39%, while maintaining a 100% PPV. These percentages were lower compared to the study performed in children where 62% could be correctly diagnosed as peanut allergic or peanut tolerant with sIgE against Ara h 2 and 80% when combining Ara h 2 and the BAT. This may be explained by the fact that in children a 100% NPV cut‐off for Ara h 2 sIgE and the BAT could be determined [2, 3], while in adults Ara h 2 cannot be used to rule out a peanut allergy.

In conclusion, combining Ara h 2‐sIgE with the BAT increases the diagnostic value in adults with suspected peanut allergy and may reduce the number of needed oral food challenges. Future studies in larger adult cohorts are needed to validate these results.

## Author Contributions

T.T.M.L., J.J.W.K., A.C.K., E.F.K., D.H., and B.A.W. designed the study. J.S.H.S. and J.J.W.K. performed the analysis. J.S.H.S., K.C.M.V., E.F.K., A.C.K., J.J.W.K., and T.T.M.L. drafted the manuscript. J.S.H.S., J.J.W.K., and T.T.M.L. wrote the final manuscript. All authors reviewed and approved the final manuscript.

## Funding

This work was supported by a grant from NWO TA PPS fund (ENPPS.TA.019.008).

## Conflicts of Interest

A.C. Knulst received institutional sponsoring for research or consultancy, outside the submitted work from: ALK, Thermofisher, Nutricia/Danone, DBV technologies, Novartis, EUROIMMUN, Stallergenes Greer.

## Data Availability

The dataset analysed during the current study is deposited in DataverseNL (doi: 10.34894). Access to the data is available upon request.
